# Difficult Intravenous Access Requiring Ultrasound in the Emergency Department: Associations With Delays in Care and Areas for Quality Improvement

**DOI:** 10.7759/cureus.44960

**Published:** 2023-09-09

**Authors:** Derrick Huang, Lucas Winter, Emily Macauley, Thomas Alterman, Bryson Renouard, James L Wilson, Leoh N Leon

**Affiliations:** 1 Emergency Medicine, University of Central Florida, Ocala, USA

**Keywords:** emergency room resuscitation, delays in care, emergency department throughput, emergency medicine, difficult intravenous access, ultrasound-guided vascular access, point-of-care-ultrasound

## Abstract

Background

Patients with difficult intravenous access (DIVA) requiring ultrasound-guided intravenous (USGIV) access have been associated with delays in treatment, imaging, and disposition in academic emergency department (ED) patient populations. Our objective in this study was to characterize differences in time to intravenous access, imaging, and disposition between patients with DIVA versus those without DIVA requiring USGIV access in a community ED while also assessing for DIVA-associated comorbidities.

Methods

A cross-sectional, observational analysis was performed on admitted ED patients evaluated from September 2 to September 31, 2022, at a community ED. Patients with DIVA were defined as patients with two failed attempts at traditional intravenous placement. These patients require USGIV placement per institutional protocol. Patients younger than 18 years of age, trauma admissions, repeated visits from the same patient, patients with missing data, and direct hospital admissions were excluded. Continuous variables were recorded with medians and included ED throughput measures of time to vascular access, contrast CT imaging, and disposition. Differences in median times between DIVA patients versus non-DIVA patients were assessed with the Mann-Whitney U-test. Categorical data involving comorbidities were reported as percentages, and differences in proportions between DIVA versus non-DIVA patients were assessed via chi-square tests. Multivariate logistic regression analysis evaluated for correlations between DIVA and times to access, contrast CT imaging, disposition, and significant covariates while adjusting for demographic information.

Results

A total of 1250 patients were included in this investigation (5.8% associated with DIVA requiring USGIV access). The median age of all subjects was 69 (interquartile range = 58, 79) with no significant difference between the DIVA and non-DIVA groups. Patients with DIVA were more likely to be female in comparison to patients without DIVA (65.3% and 51.2%, respectively, p < 0.05). Patients with a history of end-stage renal disease (ESRD) (p < 0.001), intravenous drug use (IVDU) (p < 0.001), and venous thromboembolism (p < 0.05) had statistically significant associations with DIVA. On regression analysis, patients with DIVA were more likely to have a history of ESRD with an odds ratio (OR) of 3.56 (95% confidence interval (CI): 1.62-7.81) and a history of IVDU with an OR of 14.29 (95% CI: 5.17-39.54). Patients with DIVA were associated with statistically significant greater median times to vascular access, contrast CT imaging, and disposition (p < 0.001 for time to access and disposition and p < 0.01 for time to contrast CT imaging).

Conclusion

In this study, DIVA cases requiring USGIV access were positively associated with significantly longer times to access, contrast CT imaging, and disposition compared to patients without DIVA at our community ED. Comorbidities such as IVDU and ESRD had statistically significant associations with DIVA requiring USGIV access.

## Introduction

Peripheral intravenous (PIV) catheter insertion, which occurs in an estimated 80% of patients, is a critical component of modern-day hospital care and is a necessary means of obtaining laboratory measures, contrast diagnostic imaging, and delivering life-saving therapies [1]. Quick intravenous access is of high importance in the emergency department (ED), given the need for rapid assessment, stabilization, and management of acutely ill patients. Patients with difficult intravenous access (DIVA) are relatively common, occurring in as much as one-third of patients requiring PIV access [2]. Many factors complicate the ease of PIV access, including age, race, obesity, intravenous drug use (IVDU), end-stage renal disease (ESRD), hypovolemia, chemotherapy, and a variety of other chronic medical problems [3-6]. These patients are more likely to experience delays in care, which are associated with increased mortality, worse patient satisfaction, a longer hospital stay, and disruption of the efficient throughput of patients in the ED [7-11].

Given the relative ease of execution, ultrasound-guided intravenous (USGIV) catheter placement is the next best step in obtaining intravenous access with a low threshold to implement [12]. As many as 75% of patients with DIVA ultimately require USGIV access [2]. Although USGIV access may be prone to failure from complications such as extravasation and dislodgement, this procedure is highly successful in obtaining PIV access in patients with difficult anatomy [2,13]. This reduces the need for more invasive central venous (CV) catheter insertions in the ED, avoiding risks that include infection and the need for more advanced training [14]. Patients are also more satisfied with their care when USGIV access is utilized [15,16].

Identifying determinants of delays in care in the ED is essential to implementing future quality improvements to improve health outcomes and boost patient satisfaction [7-11]. Hindrances in care associated with DIVA have been shown to be significant in studies performed at urban, academic research institutions [6,7]. As these institutions place a significant focus on research and education, studies at these institutions may not be fully generalizable to non-academic community EDs, which have different operational missions and can be associated with higher admission rates and resource utilization [17-19]. Utilizing patients with DIVA requiring USGIV access as a marker for DIVA, we sought to quantify the prevalence, risk factors, and impact of delays in care associated with DIVA in a community ED in order to highlight target areas for quality improvement and the role ultrasound may play.

## Materials and methods

Study design and setting

This was a cross-sectional, observational analysis of admitted patients receiving PIV access (including both traditional visualization with palpation and ultrasound-guided approaches) evaluated from September 2, 2022, to September 31, 2022, at Ocala Regional Medical Center, in Ocala, Florida. By standard definition, patients with DIVA are those with two failed attempts at intravenous placement using traditional visualization of veins and palpation [6]. At our ED, patients require two attempts by a registered nurse (RN) prior to USGIV placement per standard protocol. Thus, the requirement of USGIV access is used as a marker for DIVA in this study. Intravenous access requiring ultrasound is performed by emergency physicians (EPs), RNs, medics, and ED technicians after they are trained by an ultrasound fellowship-trained emergency medicine physician with a standardized didactic session. With regards to the definition of community versus academic ED in this study, our community ED does not have a primary mission of teaching, scholarly research activity by faculty physicians with grant funding, or a direct association with a medical school or university program in alignment with definitions per the Academy of Academic Administrators of Emergency Medicine/Association of Academic Chairs of Emergency Medicine [19].

In order to allow for comparison and generalization of findings from this study to other healthcare settings, we discuss the characteristics of our ED. Our institution is a non-university, community-based ED with an annual census of about 97,000 patients and an annual admission rate of 24% (including fast-track and triage patients). Approximately 10% of those admitted are dispositioned in the intensive care unit. The ED is designated as a level 2 trauma center and a comprehensive stroke center. There are two ultrasound machines with linear probes for USGIV placement. The ED has a maximum capacity of about 50 beds. This quality improvement study was exempted by our local institutional review board.

Subject selection and data acquisition

**Figure 1:**
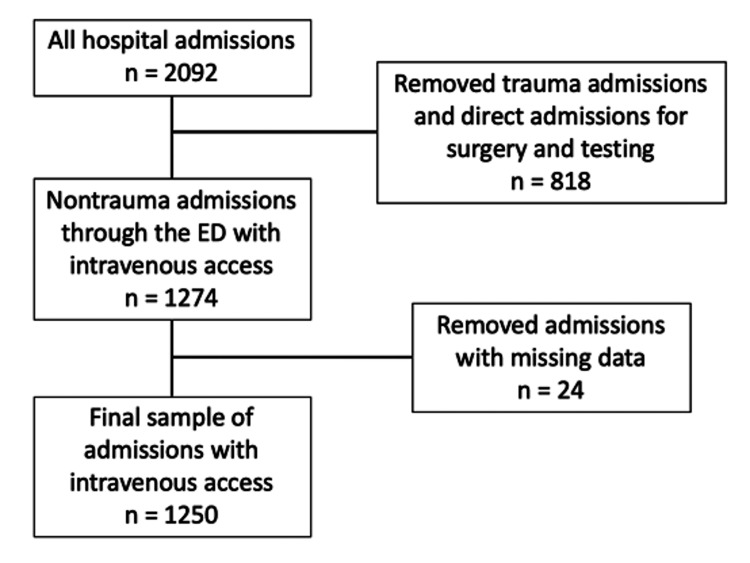
Inclusion flow diagram indicating the number of total hospital admissions during the study period and the number of patients removed due to admission to trauma service, direct hospital admission for surgery and testing, and missing data

The study included patients admitted to the ED of Ocala Regional Medical Center, in Ocala, Florida, who obtained intravenous line placement from September 2, 2022, to September 31, 2022. Patients younger than 18 years of age, trauma admissions, repeated visits from the same patient, and direct admissions to the hospital were excluded. Patients dispositioned to the trauma service and patients with direct admissions to the hospital, including visits for scheduled operations and testing, were excluded, as these patients do not necessarily require intravenous access for admission. Patients with missing data were also excluded. Applying the exclusion criteria, the final sample was composed of 1250 patients (Figure 1).

Observational data on patients admitted with intravenous access were obtained in a standardized fashion in real time. De-identified information on patients, including information on the presence or absence of specified comorbidities, was recorded for admitted patients. For admitted patients requiring USGIV access, information on the type of provider performing the procedure and the number of attempts required for success was recorded. Times of patient encounter, intravenous placement, contrast CT imaging, and disposition were recorded.

Statistical analysis

Given the non-normal distribution of the throughput data, continuous variables were recorded with medians and the 25th and 75th percentiles. Continuous variables included the median time of minutes from patient presentation (defined as the time of initial order to obtain access per protocol or physician-placed order) to vascular access, contrast CT imaging, and disposition. Non-continuous variables, including the number of attempts required for USGIV access, were reported with a mean and standard deviation. For the comparison of medians, differences were assessed with the Mann-Whitney U-test. For comparison of means, differences were assessed with the Student's t-test. Categorical data were reported as percentages, with differences in proportions assessed via chi-square tests. This categorical data included independent variables of a history of ESRD, chronic kidney disease (CKD), congestive heart failure (CHF), chronic obstructive pulmonary disease (COPD), morbid obesity, IVDU, malignant cancer, venous thromboembolism (VTE), and coronary artery disease (CAD). Multivariate logistic regression analysis with calculated odds ratios (OR) was used to evaluate for correlations between DIVA and times to access, contrast CT imaging, disposition, and significant covariates while adjusting for demographic information. All statistical analyses were performed in the IBM Statistical Package for the Social Sciences (SPSS) software version 26 (IBM Corp., Armonk, NY, USA).

## Results

A total of 1250 patients were included in the study. In this sample, 5.8% of patients had DIVA. Median values were statistically compared using the Mann-Whitney U-test. The median age of all subjects was 69 (interquartile range (IQR) = 58, 79), with no significant difference between the DIVA and non-DIVA groups, which had median ages of 67.5 (IQR = 53.5, 79) and 69 (IQR = 58, 79), respectively (p > 0.05).

On chi-square test analysis, patients with DIVA had a significant relationship with being female in comparison to patients without DIVA (65.3% and 51.2%, respectively, p < 0.05). Additionally, patients with DIVA in comparison to patients without DIVA had a significant relationship with a history of ESRD (13.9% versus 4.0%, p < 0.001), IVDU (12.5% versus 0.8%, p < 0.001), and VTE (16.7% versus 8.5%, p < 0.05). The characteristics of the subjects are summarized in Table 1.

**Table 1:** Patient characteristics and demographics DIVA: difficult intravenous access; ESRD: end-stage renal disease; CKD: chronic kidney disease; COPD: chronic obstructive pulmonary disease; IVDU: intravenous drug user; VTE: venous thromboembolism; CAD: coronary artery disease The interquartile range is displayed for age in parentheses. Percentages are displayed in parentheses for categorical variables.

Intravenous type	Total	Non-DIVA	DIVA	p-value
N	1250	1178	72	N/A
Age (years)	69 (58,79)	69 (58,79)	67.5 (53.5,79)	> 0.05
Age category (years, %)				
18-49	170 (13.6)	157 (13.3)	13 (18.1)	> 0.05
50-64	321 (25.7)	302 (25.6)	19 (26.4)	> 0.05
65+	759 (60.7)	719 (61.0)	40 (55.6)	> 0.05
Sex (% female)	650 (52.0)	603 (51.2)	47 (65.3)	< 0.05
Comorbidities (%)				
ESRD	57 (4.6)	47 (4.0)	10 (13.9)	< 0.001
CKD	170 (13.6)	158 (13.4)	12 (16.7)	> 0.05
CHF	270 (21.6)	257 (21.8)	13 (18.1)	> 0.05
COPD	255 (20.4)	239 (20.3)	16 (22.2)	> 0.05
Morbid obesity	91 (7.3)	82 (7.0)	9 (12.5)	> 0.05
IVDU	19 (1.5)	10 (0.8)	9 (12.5)	< 0.001
Cancer	134 (10.7)	122 (10.4)	12 (16.7)	> 0.05
VTE	112 (9.0)	100 (8.5)	12 (16.7)	< 0.05
CAD	364 (29.1)	347 (29.5)	17 (23.6)	> 0.05

On regression analysis comparing DIVA versus non-DIVA groups, patients with DIVA were more likely to have a history of ESRD with an OR of 3.56 (95% confidence interval (CI): 1.62-7.81) and a history of IVDU with an OR of 14.29 (95% CI: 5.17-39.54) (Table 2).

**Table 2:** Logistic regression results displaying the associations between difficult intravenous access (DIVA) status and demographic and throughput variables *Time in minutes; each variable of time was run separately on the regression model. OR: odds ratio; CI: confidence interval; ESRD: end-stage renal disease; CKD: chronic kidney disease; COPD: chronic obstructive pulmonary disease; IVDU: intravenous drug user; VTE: venous thromboembolism; CAD: coronary artery disease

Independent variable	OR (95% CI)	p-value
Age (years)		
65+	0.91 (0.45, 1.87)	> 0.05
50-64	0.83 (0.38, 1.84)	> 0.05
18-49	Reference	
ESRD		
Yes	3.56 (1.62, 7.81)	< 0.01
No	Reference	
IVDU		
Yes	14.29 (5.17, 39.54)	< 0.001
No	Reference	
VTE		
Yes	1.69 (0.84, 3.39)	> 0.05
No	Reference	
Sex		
Male	0.52 (0.31, 0.88)	< 0.05
Female	Reference	
Time to access*		
≥60	15.83 (8.13, 30.84)	< 0.001
30 to <60	9.92 (5.09, 19.33)	< 0.001
0 to <30	Reference	
Time to contrast*		
≥120 min	1.79 (1.11, 2.87)	< 0.05
0 to <120	Reference	
Time to disposition*		
≥240	5.01 (2.13, 11.80)	< 0.001
120 to <240	2.66 (1.55, 4.55)	< 0.001
0 to <120	Reference	

**Figure 2:**
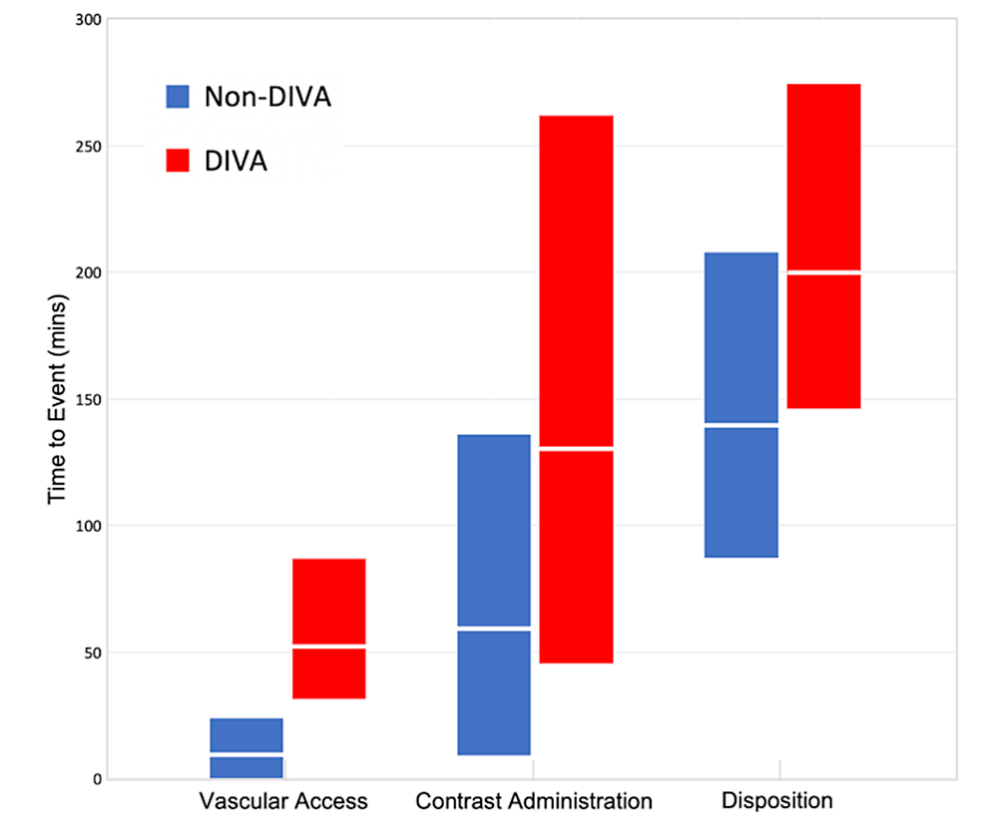
Box plot exhibiting the comparison of time to events between non-DIVA and DIVA patients The top and bottom of each box depict the 25th and 75th percentiles, while the line in between represents the 50th percentile or median. Light blue and red boxes represent non-DIVA and DIVA patients, respectively. DIVA: difficult intravenous access

Regarding the impact of DIVA on ED throughput, differences in median times of minutes from patient presentation to vascular access, contrast CT imaging, and disposition between patients with versus without DIVA status were compared and were statistically significant (p < 0.001 for time to access and disposition and p < 0.01 for time to contrast CT imaging) (Figure 2).

For the median time from patient presentation to vascular access, patients with DIVA were more likely to have delays of ≥60 minutes with an OR of 15.83 (95% CI: 8.13-30.84), and 30 to <60 minutes with an OR of 9.92 (95% CI: 5.09-19.33). For the median time from patient presentation to contrast CT imaging, patients with DIVA were more likely to have a delay of ≥120 minutes with an OR of 1.79 (95% CI: 1.11-2.87). For the median time from patient presentation to disposition, patients with DIVA were more likely to have delays of ≥240 minutes with an OR of 5.01 (95% CI: 2.13-11.80) and 120 to <240 minutes with an OR of 2.66 (95% CI: 1.55-4.55). Additionally, utilizing the Student's t-test, the mean difference in the number of attempts to achieve USGIV access between physicians and non-physicians was not statistically significant (respectively, 1.5 ± 1.0 versus 1.2 ± 0.49, p > 0.05), with 59.7% of patients with DIVA obtaining their intravenous access from physicians.

## Discussion

We investigated the association of DIVA requiring USGIV access with various throughput measures to quantify the magnitude of delay in patient care in a community ED setting. In this study population, 5.8% of patients had DIVA requiring USGIV access. In comparison, prior studies ranged from 3.1% to almost 13% of patients presenting with DIVA [7]. This may reflect true population differences; however, other studies may have different definitions of DIVA or include discharged patients. In our study population, DIVA was also associated with statistically significant and substantial delays of 42.5, 70, and 60.5 minutes in median times to vascular access, contrast CT imaging, and disposition, respectively (Figure 2). Of note, the median delay for contrast CT imaging of 70 minutes was significantly longer than the median delay for intravenous access, suggesting an additive element. A synergetic effect may exist due to the interdependency of multiple departments. For example, delays in intravenous access can disrupt the workflow of CT imaging technicians, resulting in an aggravated delay in ED throughput [20].

The findings in our investigation support the association of DIVA with delays in ED care and assessment, building upon prior studies predominantly performed at academic centers. In comparison to a related study at an academic center, overall disposition times at our community ED were approximately 40-60 minutes shorter for both DIVA and non-DIVA patients, whereas the median delay in admission associated with DIVA patients was greater at our community ED compared to the academic center (60.5 versus 37 minutes, respectively) [7]. The greater delay in disposition for patients with DIVA in the community setting may be partially attributed to a lack of available, trained personnel for USGIV placement compared to academic programs, which often have an ultrasound fellowship program with ultrasound-trained faculty [7]. However, differences in delays are also likely to be highly dependent on local patient populations and available ED resources, such as the availability of more ultrasound equipment [7].

Difficult vascular access was also associated with various comorbidities. In our study population, patients with DIVA had statistically significant, positive associations with a history of ESRD (p < 0.001), IVDU (p < 0.001), and VTE (p < 0.05) (Table 1). On regression analysis controlling for demographic information, DIVA had statistically significant associations with a history of ESRD and IVDU (Table 2). A history of IVDU has consistently been shown to have significant correlations with DIVA [6,21]. As in other studies, obesity was not associated with DIVA in our patient population; however, there are significant biases in assessing for and recording a history of obesity, which may affect interpretation [6,21]. Evidence is mixed on whether there is an association between ESRD and DIVA. Prior studies suggest ESRD is associated with DIVA, as ESRD patients often have depleted superficial veins from distortion of their vasculature secondary to chronic hemodialysis and the need for access; however, some studies do not show an association of ESRD with DIVA [6,21-23]. In this study, patients with a history of ESRD showed a significant association with DIVA. This difference may in part be due to the overall advanced age of our population in comparison to other studies, as distortion of vascular anatomy may be time-dependent.

Our study found no statistical difference in the mean number of attempts to obtain USGIV access between EPs and non-EPs. Prior studies have shown ED technicians and nurses can be trained in relatively brief didactic sessions to acquire competency in USGIV access, although baseline experience was also a factor in predicting procedural success [16, 24, 25]. Additionally, experienced nurses may have a preexisting strong grasp of identifying patients with risk factors associated with DIVA [5]. This can accelerate USGIV training and facilitate the development of throughput protocols that expedite the use of USGIV placement when traditional visualization of veins and palpation are deemed to have a high likelihood of failure. Although limited by a relatively small sample size, the data from this study underscores the effectiveness of training non-EPs to increase the pool of providers available for USGIV access. Compounding potential resource constraints, the availability of ultrasound machines may also be a significant bottleneck to establishing vascular access. Increased provider training and the number of machines available may serve as potential future quality improvement interventions.

There were several strengths and weaknesses in our investigation. In our study, DIVA cases were defined as those patients who failed two attempts by the RN and thus required USGIV access. However, this definition does not encompass the full spectrum of DIVA, which includes patients who receive vascular access outside of ultrasound-guided peripheral access, which includes the use of intraosseous (IO) access, external jugular (EJ) access, and CV access. Indeed, according to one study, only as many as 75% of DIVA patients require ultrasound-guided PIV access, specifically [2]. However, in our study, the department protocol requiring USGIV access after two failed attempts improved the validity of using USGIV access as a marker for DIVA. In addition, real-time acquisition of data and a strict vascular access protocol generally allowed for the exclusion of patients requiring other forms of advanced access. In our sample, no patients with DIVA required IO, EJ, or CV access, which is not surprising given the effectiveness of ultrasound-guided PIV placement [2,12]. Furthermore, real-time data acquisition ensured patients who received ultrasound-guided PIV access were not missed, which is possible in studies involving retrospective data mining when procedures are not logged into the medical record [7]. Despite these strengths, real-time data acquisition introduces bias during the recording of data, and it is not possible to account for all cases that occur outside of the protocol. For example, less experienced nurses may require USGIV access more often, whereas more experienced nurses may break protocol and employ USGIV access prior to visualization and palpation techniques.

Despite the significant correlations between patients with DIVA requiring USGIV placement and ED throughput measures, the cross-sectional nature of this investigation does not allow for assumptions of causation. Given the standardized format of obtaining data and biases in data acquisition, this study cannot account for all possible confounders that may affect the correlations found in this study. Standardized acquisition of comorbidities per medical history was also limited and does not account for the full spectrum of disease. For example, the severity of obesity, or CHF, was not captured. Although the goal of this study is to broaden the investigation of the role of USGIV access for DIVA patients in community EDs, which have different operational goals compared to academic centers, generalizability is still limited by numerous factors. For example, the amount and quality of ED resources, the heterogeneous nature of intradepartmental dynamics unique to every hospital, the degree of ED crowding, and seasonal variability may confound generalizability. Future studies should utilize a larger sample size over a longer period of time to better account for circumstantial variation, bias, and risk factors for DIVA. In addition, future studies can improve the analysis of possible risk factors for DIVA, such as obesity, by using body mass index and should assess the value of the degree of USGIV-trained staff and the availability of ultrasound machines in improving ED throughput.

## Conclusions

In this study, patients with DIVA requiring USGIV access were positively associated with longer times to vascular access, contrast CT imaging, and disposition compared to patients without DIVA at our community ED. Comorbidities such as IVDU and ESRD were associated with statistically significant associations with DIVA requiring USGIV access. These findings build on prior investigations at academic centers and further open up avenues for future research on the possible benefit of increased USGIV access training for non-EPs. Furthermore, there may be potential for predicting DIVA via associated risk factors and therefore employing earlier use of USGIV access.
